# Intrinsic Resting-State Activity in Older Adults With Video Game Experience

**DOI:** 10.3389/fnagi.2019.00119

**Published:** 2019-05-21

**Authors:** Hai-Yan Hou, Xi-Ze Jia, Ping Wang, Jia-Xin Zhang, Silin Huang, Hui-Jie Li

**Affiliations:** ^1^Chinese Academy of Sciences (CAS) Key Laboratory of Behavioral Science, Institute of Psychology, Beijing, China; ^2^Department of Psychology, University of Chinese Academy of Sciences, Beijing, China; ^3^Institute of Developmental Psychology, Faculty of Psychology, Beijing Normal University, Beijing, China

**Keywords:** video game experience, video game players, non-video game players, amplitude of low-frequency fluctuations, older adults

## Abstract

Playing video games is a prevalent leisure activity in current daily life, and studies have found that video game experience has positive effects in several cognitive domains. However, few studies have examined the effect of video game experience on the amplitude of low-frequency fluctuations (ALFF) among older adults. In the current study, we compared behavioral performance in the flanker task and ALFF activities of older adults, of whom 15 were video game players (VGPs) and 18 non-video game players (NVGPs). The results showed that VGPs outperformed NVGPs in the flanker task and that VGPs showed significantly increased ALFF relative to NVGPs in the left inferior occipital gyrus, left cerebellum and left lingual gyrus. Furthermore, the ALFF in the left inferior occipital gyrus and left lingual gyrus was positively correlated with cognitive performance as measured by Mini-Mental State Examination (MMSE) scores. These results revealed that playing video games might improve behavioral performance and change intrinsic brain activity in older adults. Future video game training studies in older adults are warranted to provide more evidence of the positive effects of video game experience on behavioral and brain function.

## Introduction

With age, older adults may experience declining memory, reasoning, and speed of processing as well as peripheral vision and dynamic visual acuity (Park et al., 2002; Salthouse, 2004; Muiños and Ballesteros, 2014, 2015). In addition, normal aging is associated with reduced age-related white matter integrity and gray matter volume in the caudate, cerebellum, hippocampus and the prefrontal cortex (O’Sullivan et al., 2001; Hedden and Gabrieli, 2004; Raz et al., 2005). Increasing age is also associated with a higher incidence of neurodegenerative disorders such as mild cognitive impairment (MCI), Alzheimer’s disease (AD), and Parkinson’s disease (PD; Blennow et al., 2006; Levy, 2007; Herrup, 2010; Luck et al., 2010). These changes affect the quality of daily life of older adults and aggravate the socioeconomic burden (Williams, 2005; Mitchell et al., 2010).

Several beneficial interventions have been considered to delay cognitive decline in older adults, including memory strategy training (Li et al., 2016), dancing (Coubard et al., 2011), aerobic exercise (Erickson and Kramer, 2009), music training (White-Schwoch et al., 2013) and video game playing (Toril et al., 2014; Wang et al., 2016). Of these approaches, video games have received growing attention from researchers because of their popularity and encouraging transfer effects (Zelinski and Reyes, 2009; Oei and Patterson, 2013). Increasing evidence shows that video games are beneficial to the cognitive-perceptual domain, including reaction time (Qiu et al., 2018), visual short-term memory, processing speed and attention tasks (Ballesteros et al., 2014; McDermott et al., 2014) as well as higher-level cognitive functions such as executive function, reasoning and planning (Basak et al., 2008; Oei and Patterson, 2014; Buitenweg et al., 2017; Wang et al., 2017a).

Recent studies have provided some evidence of the neural basis for superior behavioral performance in video game players (VGPs). Kühn and Gallinat (2014) found that the amount of lifetime video gaming was positively associated with cortical thickness in the left dorsolateral prefrontal cortex and left frontal eye fields. Other studies have shown significantly larger gray matter volume in the right posterior parietal cortex in VGPs compared with non-video game players (NVGPs; Tanaka et al., 2013). Another study reported that video game experience altered the visual cortical network (Granek et al., 2010). Moreover, Bavelier et al. (2012) found that VGPs showed less recruitment of the frontoparietal attentional network during a visual search task compared to NVGPs, while VGPs showed increased activation in frontoparietal regions in a flanker task (Wang et al., 2017b). In recent resting-state functional magnetic resonance imaging (fMRI) studies, Gong et al. (2015) found that VGPs showed increased functional connectivity between the attentional and sensorimotor networks, and they further found that VGPs had enhanced functional integration both within and between the salience network and the central executive network (Gong et al., 2016).

By studying spontaneous fluctuations in blood oxygen level-dependent contrasts, resting-state fMRI studies can provide valuable insights into investigating the differences between VGPs and NVGPs (Biswal et al., 1995; Fox and Raichle, 2007). To assess spontaneous brain activity in attention deficit hyperactivity disorder, Zang et al. (2007) proposed studying the amplitude low-frequency fluctuations (ALFF), which is defined as the square root calculated at each frequency of the power spectrum within a specific low-frequency band. Mennes et al. (2011) demonstrated that resting-state ALFF had robust predictive value for behavior. Wei et al. (2012) demonstrated that individual differences in ALFF could predict the conceptual processing capacity of subjects. These findings suggested that the assessment of ALFF could be used to investigate the neural basis of video game experience in older adults.

To the best of our knowledge, no resting-state fMRI study has examined the ALFF difference between VGPs and NVGPs in older adults. In the current study, we mainly aimed to investigate the ALFF differences between older VGPs and older NVGPs in combination with behavioral performance and sought to determine whether the differences in ALFF were associated with video game experience. We hypothesized that older VGPs would present better behavioral performance and increased ALFF activity in comparison with older NVGPs.

## Materials and Methods

### Participants

Older adult volunteers were recruited by means of posters and online advertisements. All participants were healthy, had normal or corrected-to-normal vision and had more than 6 years of education. In addition, the inclusion criteria for all participants included having no medical disorders, no neurological disease, and no metallic implants in the body that could interfere with or cause injury due to the magnetic field. Participants were divided into two groups: those with video game playing experience, classified as VGPs, had played video games at least 2 h per week (more than 0.5 h per day and at least 4 days) over the previous 6 months; the time that VGPs dedicated to game-playing was approximately 395 h per year on average. Participants with no video game experience in their lifetimes were classified as NVGPs.

Thirty-seven participants participated in a resting-state fMRI scan, and four participants (two VGPs and two NVGPs) were excluded due to poor registration between functional and structural images or excessive head motion. Ultimately, 15 VGPs (nine females, median = 65.20 years, range from 55 to 74 years old) and 18 NVGPs (six females, median = 65.06 years, range from 55 to 78 years old) were included for further analysis, and no significant gender difference was found between the two groups (*χ*^2^ = 2.347, *p* = 0.126). The Mini-Mental State Examination (MMSE; Folstein et al., 1975) and the activities of daily living scale (ADL; Katz et al., 1963) were used to assess all participants. Moreover, we also investigated the hobby hours of every participant, which included calligraphy, chess, playing cards and other hobbies other than video games.

The study was approved by the local ethics committee of the Institute of Psychology, Chinese Academy of Sciences (IPCAS). We obtained written informed consent from all participants prior to the study.

### Data Acquisition and Analysis

#### Scanning Procedure

Magnetic resonance imaging scans were conducted on a General Electric 3T scanner (GE Discovery MR750). Plastic earplugs were used to reduce scanner noise. The participants were instructed to lie quietly with their eyes closed and to keep their head as still as possible. Resting-state functional images were acquired using gradient-echo sequences with the following parameters: repetition time (TR) = 2,000 ms, echo time (TE) = 30 ms, flip angle = 90°, the number of slices = 43, slice thickness = 3.5 mm, slice gap = 0.5 mm, voxel size = 3.75 × 3.75 × 3.5 mm^3^, field of view (FOV) = 240 × 240 mm^2^, matrix size = 64 × 64, axial orientation. A T1 structural image was recorded using spoiled gradient echo sequences with the following settings: TR = 39.9 ms, TE = 24.76 ms, flip angle = 10°, the number of slices = 256, slice thickness = 1 mm, voxel size = 1 × 1 × 1 mm^3^, FOV = 256 × 256 mm^2^, sagittal orientation. Task-based fMRI images were acquired after the resting-state functional images and T1 structural images. All participants performed the flanker task during the scanning, and the task-based fMRI results were reported elsewhere (Wang et al., 2017b). The flanker paradigm consisted of strings of arrows, and participants were required to focus on the central arrow and to ignore surrounding arrows that could be congruent (e.g., >>>>>) or incongruent (e.g., <<><<) to the central arrow. The participants used the number button “1” or “4” on the response pad to indicate the left or right direction, respectively, of a central arrow after a fixation cross was presented, and their reaction time and accuracy were recorded. More details can be found in our previous study (Wang et al., 2017b). The flanker task performances were used as behavioral performance in the current study.

#### Data Preprocessing

Resting-state fMRI data were carried out using RESTplus V1.2^1^ based on MATLAB2014a. Preprocessing included the following steps: (1) Discarding the first 10 time points to allow the tissue to reach steady state magnetization and to allow participants to adapt to the scanning noise; (2) Slice timing to temporally correct the interleaved slice acquisition; (3) Aligning each volume to the mean EPI image to correction between head movements; (4) Normalizing using T1 image unified segmentation; (5) Spatial smoothing with a 6 mm full width at half maximum Gaussian kernel; (6) Removing the linear trend within the time series; (7) Regressing out the nuisance covariates, which included 6 head motion parameters, a global mean signal, a white matter signal and a cerebrospinal fluid signal; and (8) Temporal filtering (0.01–0.1 Hz) was performed on the time series of each voxel to reduce the effect of low-frequency drifts and high-frequency noise.

#### ALFF Calculation

The filtered time series was converted to the frequency domain using the Fast Fourier Transform. Then, the square root of the power spectrum at each frequency was averaged across a frequency band of 0.01–0.1 Hz. This averaged square root was taken as the ALFF, and the result of normalized ALFF values of each participant was adopted for statistical analysis. The procedure for calculating ALFF has been described in previous studies (Yang et al., 2007; Zang et al., 2007).

#### Statistical Analysis

A two-sample *t*-test was conducted on the ALFF between VGPs and NVGPs. Multiple comparisons were corrected using familywise error correction (FWE) based on Gaussian random-field theory, which yielded a voxel level of *p* < 0.01 and a cluster level of *p* < 0.05.

For the flanker task, reaction time from incorrect trials was excluded, and the difference in the average reaction time in the congruent condition subtracted from that in the incongruent condition was used as behavioral performance. Considering the relatively small sample size in each group, we used the Mann-Whitney *U* test to compare the demographic variables and flanker task performances between older VGPs and NVGPs with SPSS 19.0.

#### Correlation Analyses

To investigate the relationship between the ALFF values and the behavioral measures, including hobby hours, MMSE scores, and flanker task performance, we used the brain regions with significant differences in the two-sample* t*-test as regions of interest (ROI), then extracted the signals from the ROI of each participant and computed the Pearson’s correlation coefficients between ROI signals and behavioral performance for all participants.

## Results

The results showed that the two groups did not differ in age, education years and ADL. However, Older VGPs showed significant higher score in MMSE and more hobby hours than older NVGPs (Table 1). For flanker task performance, the mean reaction time of the VGPs was significantly shorter than that of the NVGPs (*U* = 192, *Z* = 2.06, *p* = 0.04). In addition, the effect size reached 0.816, which was considered large (Cohen, 1988).

**Table 1:** Demographic information on video game player (VGP) and non-video game player (NVGP) participants.

	VGP (*N* = 15)		NVGP (*N* = 18)		*Z*	*p*
	M (SD)	Range	M (SD)	Range		
Age (years)	65.20 (5.92)	55–74	65.06 (7.17)	55–78	−0.16	0.87
Education (years)	13.13 (2.92)	9–18	11.78 (2.82)	9–17	−1.38	0.19
MMSE	28.87 (1.64)	25–30	27.72 (1.60)	24–30	−2.28	0.02
ADL	8.13 (0.35)	8–9	8.00 (0.00)	8–8	−1.57	0.53
Hobby hours	225.80 (163.99)	7–560	125.11 (211.56)	0–843	−2.54	0.01

*Note: N, sample number; MMSE, Mini-Mental State Examination; ADL, Activities of Daily Living Scale; Hobby hours, total hours spent on hobbies other than video games*.

### Group Differences in ALFF

**Figure 1:**
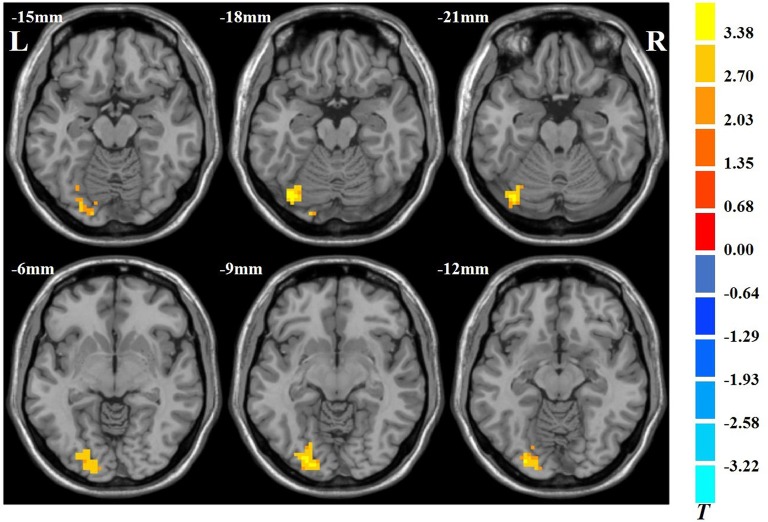
Increased spontaneous amplitude of low-frequency fluctuation (ALFF) activity of video game players (VGPs) in comparison with non-video game players (NVGPs).

The results showed that VGPs showed increased ALFF in the left inferior occipital gyrus, left cerebellum, and left lingual gyrus compared with NVGPs (Figure 1, Table 2). No decreased ALFF regions were found in VGPs compared with NVGPs. However, when MMSE, hobby hours, gender, and years of education were controlled as covariates, the significant results disappeared.

**Table 2:** Regions showing ALFF differences between VGPs and NVGPs.

Brain regions	Peak MNI coordinates				Peak *T*-value
	Voxels	*x*	*y*	*z*	
Left IOG	38	−27	−87	−9	3.79
Left cerebellum	30	−36	−78	−21	4.06
Left LG	29	−27	−90	−12	3.48

*Note: x, y, and z are coordinates of primary peak locations in the MNI space. T is the statistical peak value of the voxel showing ALFF differences between VGPs and NVGPs (positive values: VGPs > NVGPs), IOG, inferior occipital gyrus, LG, lingual gyrus. Voxel level: *p* < 0.01, cluster level: *p* < 0.05, corrected for FWE*.

### Correlation Analysis

**Figure 2:**
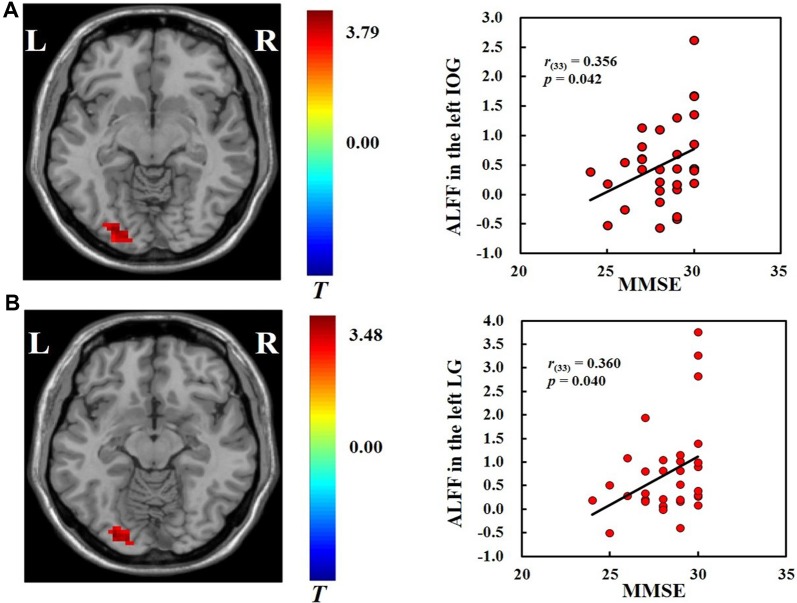
Correlations between ALFF in the left inferior occipital gyrus **(A)** and left lingual gyrus **(B)** and mini-mental state examination (MMSE) scores.

We found positive correlations between MMSE scores and the ALFF in the left inferior occipital gyrus (*r*_(33)_ = 0.356, *p* < 0.05) and left lingual gyrus (*r*_(33)_ = 0.36, *p* < 0.05; Figure 2) across all participants. No significant correlations were found between ALFF and hobby hours or flanker task performance. We also did not find significant correlations between ALFF and the behavioral measures in the VGP and NVGP groups separately.

## Discussion

In this study, we investigated behavioral performance and differences in spontaneous brain activity by measuring the ALFF of resting-state fMRI signals between older VGPs and older NVGPs. We found that older VGPs outperformed older NVGPs in the behavioral task and presented increased ALFF in the left inferior occipital gyrus, left cerebellum and left lingual gyrus compared with NVGPs.

Older VGPs showed significantly better performance in the flanker task than older NVGPs. During the flanker task, participants needed to ignore the peripheral arrows while concentrating on the center arrows, and executive control was required to identify whether the targets and distractors were in conflict. This result was consistent with previous findings that VGPs have better performance than NVGPs in the flanker task (Green and Bavelier, 2003). VGPs have also responded more quickly than NVGPs in an attention network test (Dye et al., 2009). Other studies have also shown that video game training enhances cognitive control and selective visual attention in older adults (Anguera et al., 2013; Belchior et al., 2013). In addition, a recent study found that higher-order executive function skills were improved after training on a physics-based puzzle game (Oei and Patterson, 2014). VGPs have an advantage in top-down distractor suppression (Chisholm and Kingstone, 2012), which may be the underlying mechanism of superior performance in executive control.

VGPs showed increased ALFF in the left inferior occipital gyrus and left lingual gyrus. In our recent study, we found that VGPs compared with NVGPs showed increased activation in the left lingual gyrus in the flanker task (Wang et al., 2017b). Functional imaging studies have indicated that the amount of lifetime video game playing is positively associated with occipital gray matter volume (Kühn and Gallinat, 2014). The occipital lobe and lingual gyrus have been reported to be involved in visual processing (Cai et al., 2017). Several studies have found that the enhancement of visual function, including visuospatial attention (Green and Bavelier, 2006), contrast sensitivity (Li et al., 2009), and visual search (Castel et al., 2005), is associated with video game experience. Jung et al. (2014) revealed that a larger volume of lingual gyrus predicted better performance in neuropsychological tests. An ERP study showed increased amplitudes in the occipital cortex after playing video games for 10 h (Wu et al., 2012). We also found that VGPs showed increased ALFF in the left cerebellum in comparison with NVGPs. Recent studies have substantiated that gray matter volume in the cerebellum increases significantly in the experimental group after playing a video game for 2 months (Kühn et al., 2014). The cerebellum has been considered to contribute to motor control (Manto et al., 2012). In a clinical study, it has been reported that video game exercises are beneficial for subjects’ balance control (Betker et al., 2006).

In the current study, we found positive correlations between ALFF values in the left inferior occipital gyrus and left lingual gyrus with MMSE scores. The results suggest that increased ALFF activity is associated with better cognitive function. Previous studies have shown that older adults have cognitive and brain plasticity (Park and Bischof, 2013; Brehmer et al., 2014). The scaffolding theory of aging and cognition suggests that the adaptive aging brain engages in compensatory scaffolding in response to the challenges posed by declining neuronal structure and function (Park and Reuter-Lorenz, 2009; Reuter-Lorenz and Park, 2014). Video game playing is considered an effective way to bring about broad cognitive transfer (Zelinski and Reyes, 2009; Boot et al., 2011; Oei and Patterson, 2013; Toril et al., 2014). When immersed in video games, older VGPs need to engage more cognitive resources, and this may help them construct compensatory scaffolding due to changing visual stimulation and repetition experiences, enhancing their brain plasticity and protecting them against the cognitive decline caused by aging.

Although we speculate that older VGPs construct compensatory scaffolding to underlie cognitive and neural plasticity, the underlying biological mechanisms of ALFF activity are not clear. A recent study revealed significant positive correlations between ALFF and gamma-aminobutyric acid (GABA; Nugent et al., 2015). GABA is a main inhibitory neurotransmitter in the central nervous system (Paulsen and Moser, 1998). GABA modification plays an important role in cortical plasticity (Bütefisch et al., 2000; Ziemann et al., 2001). In the current study, we observed increased ALFF activity in older VGPs, which might be the consequence of GABA modification and increased GABA in the brain. The increased GABA in older VGPs may lead to inhibitory signaling and therefore improve inhibition ability; consequently, it may help older VGPs achieve better performance in the flanker task.

There are some limitations in the current study. The genre of the video games may be a factor that impacts diversified cognitive abilities. We could not analyze this factor due to the relatively small sample size. The current study was a cross-sectional study, and we could not exclude the contribution of other variables to the increased behavioral performance and brain activity. Future video game training studies, especially studies focusing on certain game genres, are warranted to further reveal the video game training-related effects on behavioral performance and brain activity. Moreover, demographic variables might influence the ALFF results. When we controlled for the MMSE, hobby hours, gender, and years of education as covariates, we could not observe significant ALFF results between VGPs and NVGPs; therefore, this finding has to be treated cautiously. In the future, when increasingly older adults fulfill the inclusion criteria of VGPs and meet the magnetic resonance imaging scanning criteria, researchers could recruit large numbers of participants and further reveal the video game experience-related effects on behavioral and neural changes.

## Conclusion

The present study revealed that VGPs outperformed NVGPs in the flanker task and that they present increased ALFF in the left inferior occipital gyrus, left cerebellum and left lingual gyrus compared with NVGPs. These results suggest the positive effects of video game playing on brain plasticity in older adults, suggesting that video games may be a promising tool to delay cognitive decline in older adults. Future studies are recommended to train video game naive participants and investigate ALFF effects over time.

## Author Contributions

H-JL and H-YH conceived the idea and wrote the manuscript. X-ZJ guided the data analysis. PW, SH and J-XZ collected the data and contributed towards writing the article.

## Conflict of Interest Statement

The authors declare that the research was conducted in the absence of any commercial or financial relationships that could be construed as a potential conflict of interest.

## Footnotes

^1^http://www.restfmri.net
